# Trends of Acute Hepatitis B Notification Rates in Eastern China from 2005 to 2013

**DOI:** 10.1371/journal.pone.0114645

**Published:** 2014-12-12

**Authors:** Zhifang Wang, Yaping Chen, Jinren Pan

**Affiliations:** Department of Immunization Programme, Zhejiang Provincial Center for Disease Control and Prevention, Hangzhou, Zhejiang Province, China; The Australian National University, Australia

## Abstract

Zhejiang Province was a high endemicity for hepatitis B disease in the 1990's. A number of measures implemented since then have begun to control and prevent hepatitis B. In 1992, hepatitis B vaccine came on the market. In 2002, hepatitis B vaccine was included in the national Expanded Programme on Immunization (EPI). Between 2007 and 2010, catch-up vaccination was implemented for children under 15. Since 2010, vaccination guidelines for high-risk groups have also been adopted. This study evaluated the impact of these control and prevention strategies on acute hepatitis B notification rates from 2005 through 2013. Data from the National Notifiable Disease Reporting System (NNDRS) revealed a steady downward trend in notification rates of acute hepatitis B. The most dramatic decline occurred among pre-adults, highlighting the benefits of EPI's policy of universal vaccination for children. However, the highest notification rates occurred among young adults of lower socio-economic status. These findings indicate the strong need to vaccinate young adults at risk for HBV infection as well as to collect risk-factor information in the NNDRS for monitoring and following up persons with acute hepatitis B.

## Introduction

An estimated 120 million Chinese are hepatitis B surface antigen (HBsAg)-positive carriers, which means approximately one-third of the world's hepatitis B carriers live in China [1]–[2]. An estimated 4 million of these live in Zhejiang Province [3]. In 1992, the Chinese national hepatitis B epidemiological survey determined that, in the general population in Zhejiang Province, the rate of HBsAg positivity was 11.7% [4]–[5]. This rate was significantly greater than the national average rate of 9.8% [6]–[7]. The same survey showed the highest reported incidence (>10%) of HBsAg among children aged 1–14 years [4]–[5].

These findings indicated that Zhejiang Province was a high endemicity area of HBV infection with two major transmissions. First, during infancy, infected mothers pass the infection to their newborns. Second, during early childhood, infected children pass the infection to those children who are without adequate immunity [6]–[7]. Since then, the following HBV control and prevention measures have been taken:

1992 Hepatitis B vaccine was introduced into China.

1994 All pregnant women were required to be tested for HBsAg during prenatal visits or at the time of delivery. Immunoprophylaxes were provided for infants born to HBV-infected mothers, including hepatitis B immune globulin and hepatitis B vaccine.

2002 Hepatitis B vaccine was integrated into the National Expanded Programme on Immunization (EPI).

2005 Routine immunization was administered to all infants, with emphasis on neonates uptaking a timely birth dose of Hepatitis B vaccine (24 hours after birth), followed by two additional doses at the end of the first and sixth month of age, respectively.

2007–2010 Catch-up strategies targeting all previously unvaccinated children aged ≤15 years was adopted as a supplement to routine infant vaccination.

2010 Hepatitis B vaccination was recommended to six high-risk groups, including health care workers, people who inject drugs, people who closely contact with HBsAg-positive persons, people with high-risk sexual behavior, people who frequently require blood or blood products, and hemodialysis patients. A three-consecutive-dose vaccination schedule was recommended, with the first shot at any given time, and the other two at the first and the sixth month after the first dose (0-1-6).

This study was conducted to assess the impact of these intervention efforts on acute hepatitis B notification rates obtained from the existing National Notifiable Disease Reporting System (NNDRS). The main aim of this study was to describe the secular trend of acute HBV infection notification rates in Zhejiang Province from 2005 through 2013, thus giving focus to the future immunization strategies or activities of prevention and control.

## Methods

### Introduction of the NNDRS

Since 2005, mandatory reporting of acute hepatitis B cases in China has been accomplished via the NNDRS under the Infectious Diseases Notification Regulation. The NNDRS is a web-based of reporting infectious diseases. The system promotes coordination among local health departments and among Centers for Disease Control and Prevention (CDCs) at prefectural, city, provincial, and national levels.

Physicians or health care workers manually or electronically enter acute hepatitis B cases into the NNDRS. They are required to report clinically diagnosed acute HBV infection cases (with or without laboratory confirmation) to their local CDC. They are also required to report acute hepatitis B cases to provincial and Chinese CDCs via the NNDSS within 24 hours after confirmation of diagnoses.

Physicians and health care workers have a good compliancy record with the national diagnostic criteria. First, national diagnostic criteria are available for the clinical diagnosis of acute hepatitis B. Second, training programs for them were implemented, introducing case definition and the surveillance system. Third, they have a good level of awareness with respect to reporting acute hepatitis B.

### Case definition for acute hepatitis B

The national diagnostic criteria for viral hepatitis B were launched throughout the whole country in 1995 and amended in 2008. In clinical practice, however, the newly launched case definition for acute hepatitis B (WS 299-2008) did not substantially differ from the criteria used before (GB 15900-1995). Acute hepatitis B cases were then and are now reported according to meet at least one of the following eight criteria:

1. Fatigue accompanied by nausea, vomiting, diarrhea, anorexia, and/or jaundice, together with negative HBsAg <6 months ago and currently positive HBsAg.
2. Fatigue accompanied by nausea, vomiting, diarrhea, anorexia, and/or jaundice, together with positive immunoglobulin M antibody against hepatitis B core antigen (IgM anti-HBc) and currently positive HBsAg.
3. Fatigue accompanied by nausea, vomiting, diarrhea, anorexia, and/or jaundice, together with histologic evidence of acute viral liver inflammation and currently positive HBsAg.
4. Fatigue accompanied by nausea, vomiting, diarrhea, anorexia, and/or jaundice, together with previous positive HBsAg, negative HBsAg and positive antibodies against hepatitis B surface protein (anti-HBs) during the resolved infection period.
5. Abnormal liver function test results together with negative HBsAg <6 months ago and currently positive HBsAg.
6. Abnormal liver function test results together with positive HBsAg and the current positive value of IgM anti-HBc≥1∶1000.
7. Abnormal liver function test results together with positive HBsAg and the current histologic evidence of acute viral liver inflammation.
8. Abnormal liver function test results together with previous history of positive HBsAg and the current negative HBsAg and positive anti-HBs during the resolved infection period.

### Data resources and data analysis

In this study, a dataset of acute hepatitis B cases on age, gender, occupation, and region distribution was obtained from the NNDRS. This dataset includes cases with referral dates between January 1, 2005 and December 31, 2013 in the NNDRS.

Notification rates were calculated per 100,000 persons. Age groups were defined as 0–19 years, 20–39 years, 40–59 years, and ≥60 years. For calculating regional rates, Zhejiang Province was categorized by its 11 prefecture-level cities. Occupations were chosen from the Chinese Standard Classification of Occupations 1995: (1) Farmers, fishers, and members of the working class; (2) Commercial staff; (3) Leading cadres; (4) Health personnel and teachers; and (5) Others. This study abbreviates these as five respective classes: (1) Worker; (2) Commercial; (3) Cadre; (4) Professional; and (5) Other.

Every month, health care workers in county vaccination clinics report the number of vaccinated children to the National Immunization Programme Surveillance System via city and provincial CDCs. An infant who has received the first dose of hepatitis B vaccine within 24 hours after born is defined as having been vaccinated in a timely manner. In this study, hepatitis B vaccine coverage refers to the percentage of timely vaccination.

The ethics committee of the Zhejiang Provincial CDC approved this study. Because data were analyzed and reported anonymously, there were no participants from which to request written informed consent.

Data were exported via the NNDRS to Microsoft Office Excel 2007, where duplicate and misreported cases were removed. For example, because health care workers are not required to report HBsAg-positive carriers or portable cases, when these cases show up in the list, they are considered “misreported.” The data were analyzed using SPSS (SPSS, Inc., Chicago, IL, USA) and figures were produced by Excel 2007.

The reported rate (R) of acute hepatitis B cases was calculated by dividing the number of reported acute hepatitis B cases (C) via the NNDRS by the number of inhabitants (I) registered in local public health facilities (R = C/I).

The reported timely rate (T) of hepatitis B vaccination was calculated by dividing the number of doses (D) administered within 24 hours of birth by the number of children (C) registered for vaccination in health care facilities (T = D/C).

## Results

### Overall trend of acute HBV reported incidence

**Figure 1:**
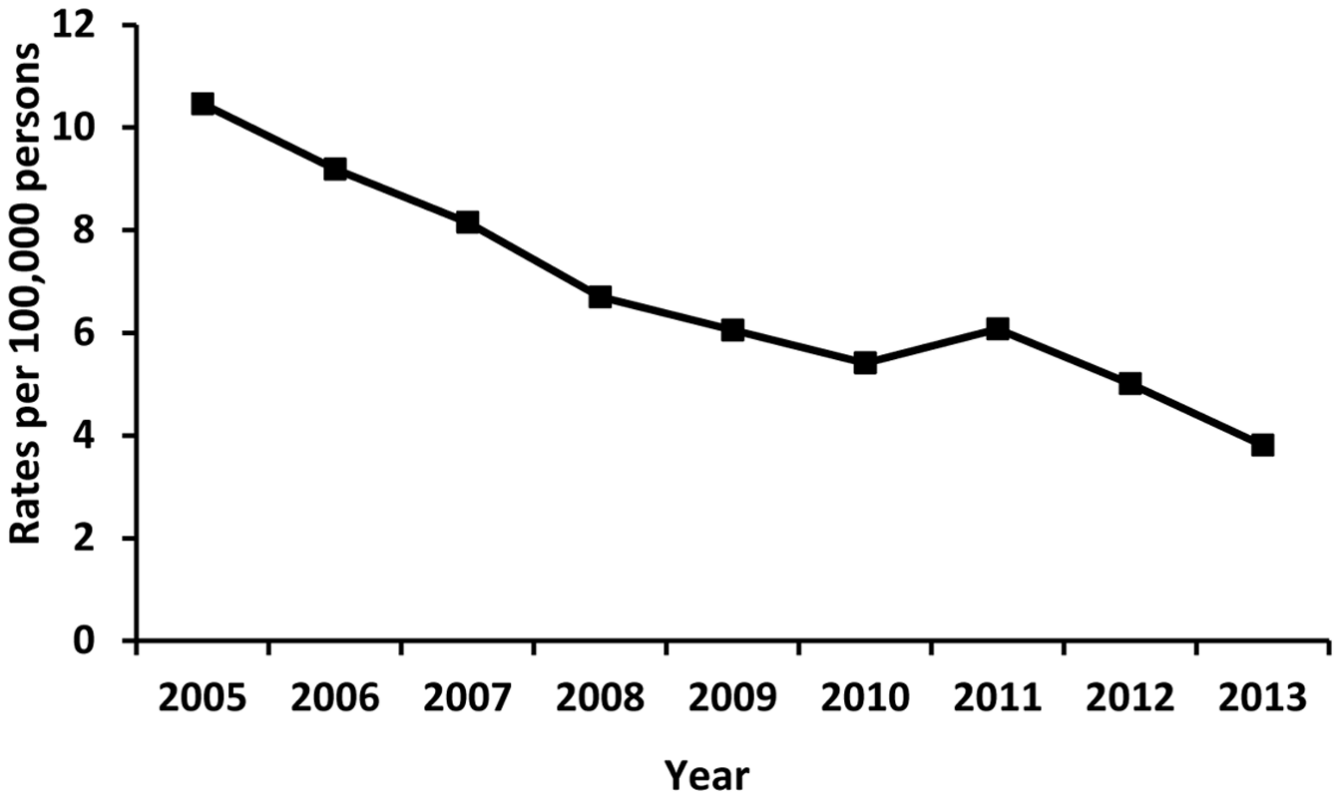
Reported rates of acute hepatitis B cases, 2005–2013.

The trend of the acute hepatitis B notification rate was followed over a period of nine years, from 2005 to 2013. The notification rate decreased significantly by almost 65%. That is, in nine years, cases per 100,000 population decreased from 10.22 in 2005 to 3.68 in 2013 (Fig. 1). Between 2005 and 2013, the acute hepatitis B notification rate showed a steady decrease. The only exception was a slight increase in 2011.

### Age distribution

**Figure 2:**
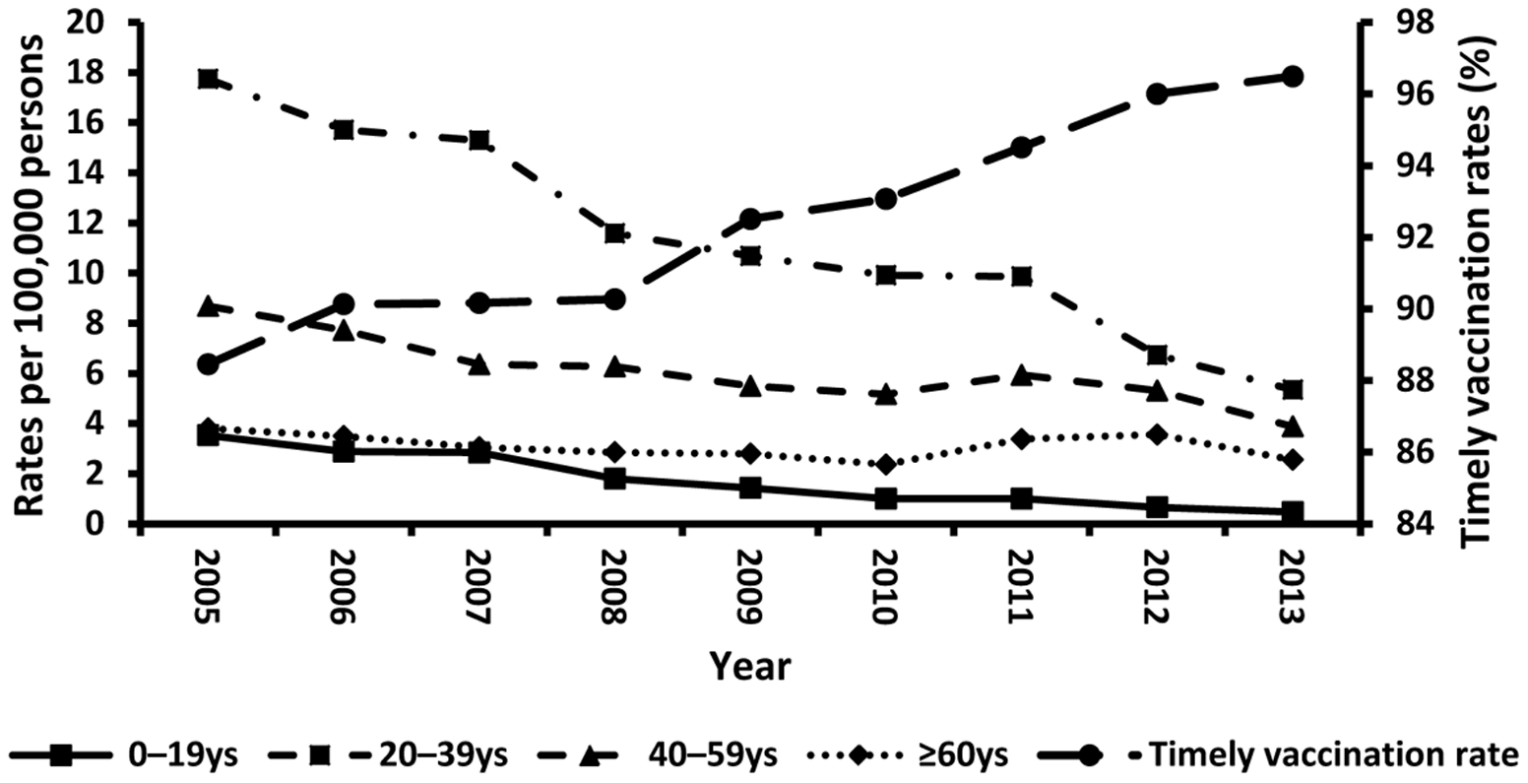
Reported rates of acute hepatitis B cases by age group and timely vaccination rates, 2005–2013.

Notification rates by age group and year are presented in Fig. 2.

Notification rates were analyzed separately for each age group in order to assess the various effects of the immunization policy on the various groups. For example, because the infant vaccination policy was initiated in 1992, the notification rate in 2013 among those younger than 20 years could roughly represent the intervention result of the infant vaccination policy for that group.

Notification rates of acute hepatitis B among all age groups declined steadily from 2005 to 2013. These notification rates decreased by approximately 90% among 0–19 year-olds, 70% among 20–39 year-olds, 55% among 40–59 year-olds, and 30% among ≥60 year-olds. By 2013, the highest notification rate per 100,000 population was among young adults aged 20–39 years (5.35 cases) while the lowest rate was among pre-adults aged ≤19 years (0.47 cases).

Timely vaccination rates are also shown in Fig. 2. In 2005, 88.45% of infants were immunized within 24 hours of birth. These timely rates held steady at 90% from 2006 to 2008, increased from 2009 to 2012, and have maintained at>95% since then.

Timely vaccination rates show an inverse relationship to notification rates. That is, from 2005 to 2013, while timely vaccination rates were increasing, notification rates were decreasing at a similar pace. Respective notification rates in 2005 for those aged 0–19, 20–39, 40–59, and ≥60 years were 3.54, 17.74, 8.68, and 3.81 cases per 100,000 inhabitants. Yet by 2013, these rates had fallen to 0.47, 5.35, 3.88, and 2.55 cases per 100,000 – respective declines of 86.78%, 69.86%, 55.28%, and 32.94%. This falling trend was strongest for the age group of 0–19 years. From 2005 to 2008, the rate for this group fell by almost 50%; then from 2008 to 2013 by approximately 75%.

### Gender distribution

For each year between 2005 and 2013, the notification rate was approximately twice as high among males as among females (Table 1). From 2005 to 2013, notification rates steadily declined for both males and females (S1 Figure). The rate for males dropped from 14.84 cases per 100,000 persons in 2005 to 4.84 cases per 100,000 in 2013, while the rate for females decreased from 5.43 cases per 100,000 in 2005 to 2.46 cases in 2013. This decrease was more extreme among males (67.38%) than among females (54.74%).

**Table 1:** Reported rates of acute hepatitis B cases by gender, 2005–2013.

	Population		Cases		Reported rates (%)	
Year	Male	Female	Male	Female	Male	Female
2005	24,829,460	23,905,853	3,684	1,299	14.84	5.43
2006	24,845,562	24,134,403	3,157	1,211	12.71	5.02
2007	25,344,319	24,455,678	2,937	1,031	11.59	4.22
2008	25,736,889	24,863,123	2,417	900	9.39	3.62
2009	26,026,789	25,173,188	2,239	813	8.60	3.23
2010	26,316,565	25,483,454	1,951	809	7.41	3.17
2011	27,965,806	26,461,027	2,265	971	8.10	3.67
2012	28,001,994	26,627,997	1,850	807	6.61	3.03
2013	28,180,006	26,590,003	1,364	654	4.84	2.46

**Figure 3:**
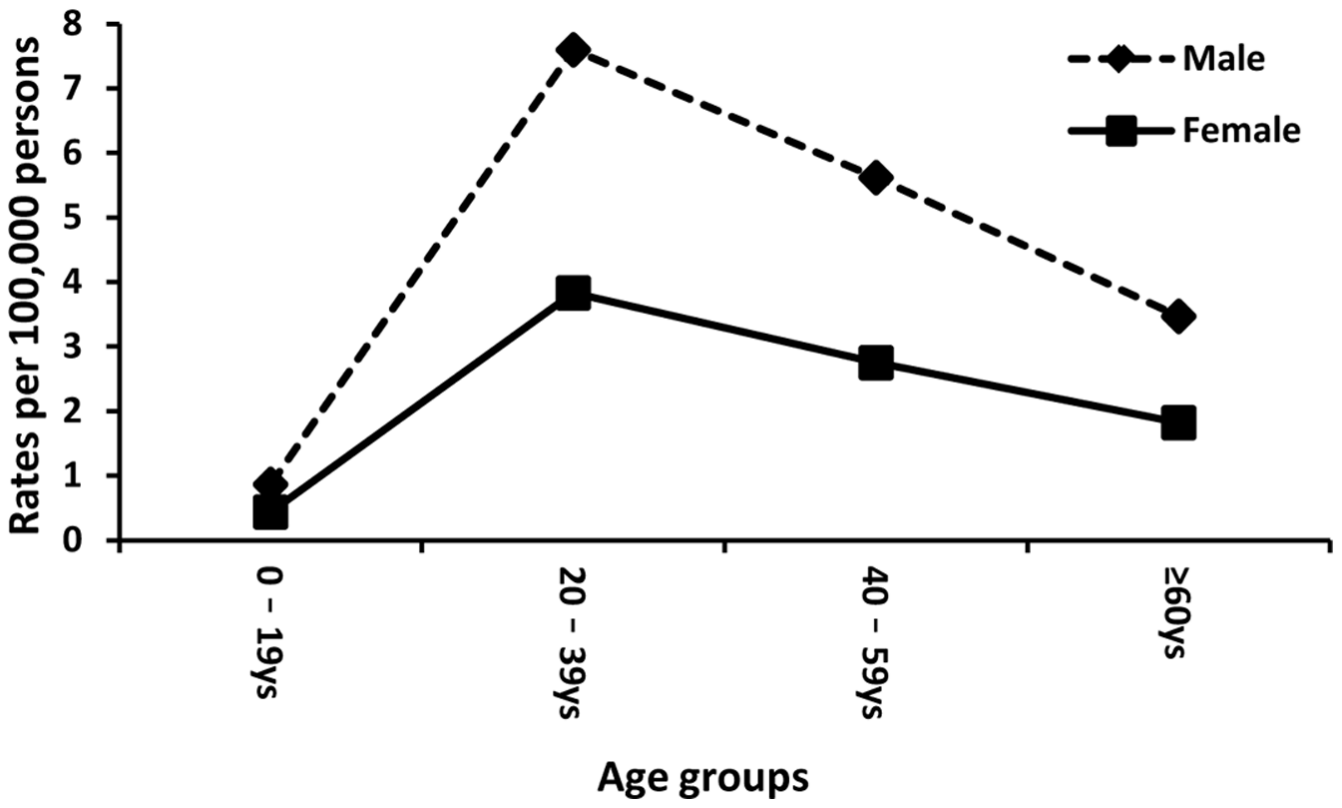
Reported rates of acute hepatitis B cases by gender and age group in 2013.

Notification rates of acute hepatitis B by gender and age group in 2013 are presented in Fig. 3. By 2013, for both boys and girls, reporting rates during childhood and adolescence had fallen to low levels. The gender gap in notification rates, however, widened between those aged 0–19 and those aged 20–39. Yet this gap narrowed between those aged 40–59 and those aged ≥60. The highest difference between men and women appeared in the age group 20–39 years, with a gender gap of 3.58 cases per 100,000 persons. The rate in this group was approximately twice as high among males (7.34 cases) as among females (3.76 cases).

### Occupational distribution

Every year, the Worker class makes up the huge majority of HBV cases (Table 2). The Worker percentage seems to tower over the percentages of the other classes: Worker, 72.67%; Commercial, 4.96%; Cadre, 3.86%; Professional, 1.77%; and Other, 16.74%. Furthermore, the percentage of the Worker class climbs from 72.02% in 2005 to 77.45% in 2013. Between 2005 and 2013, the constituent ratio among worker males (50.8%) was significantly higher than worker females (20.8%) and other occupation groups (S1 Table), with P value was less than 0.001 and χ^2^ value was 165.

**Table 2:** Composition ratios of reported acute hepatitis B cases by occupation, 2005–2013.

Year	Worker	Commercial	Cadres	Personnel	Other	Total
2005	72.02	3.65	4.13	2.07	18.12	100
2006	71.34	4.51	3.71	1.79	18.66	100
2007	72.00	4.31	3.63	1.84	18.22	100
2008	73.83	4.73	3.32	1.60	16.52	100
2009	72.08	4.46	4.36	1.51	17.60	100
2010	74.35	4.46	3.19	1.56	16.45	100
2011	71.54	6.86	4.36	2.26	14.99	100
2012	72.30	7.34	3.99	1.77	14.60	100
2013	77.45	6.14	4.01	1.09	11.30	100
2005–2013	72.67	4.96	3.86	1.77	16.74	100

**Figure 4:**
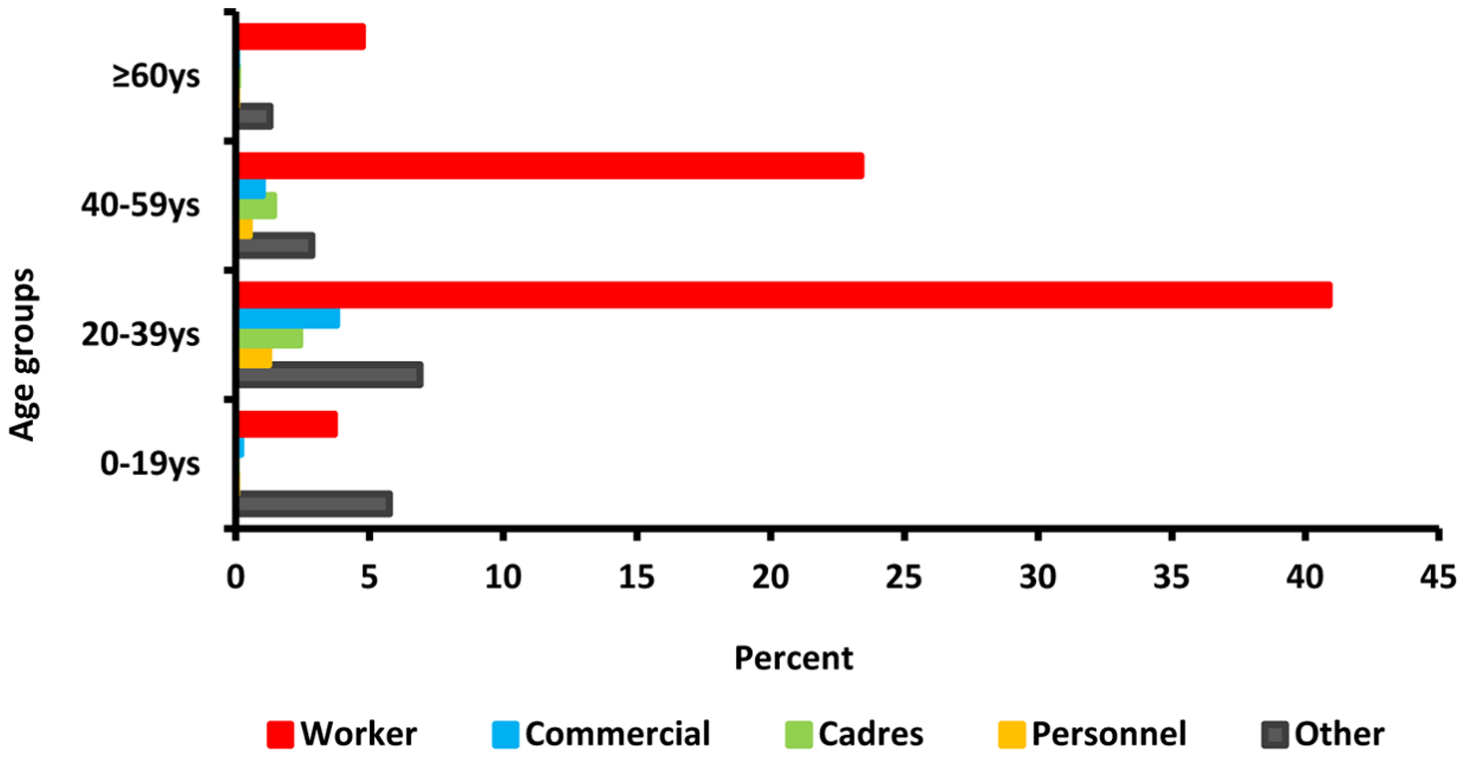
Cumulative ratios of reported acute hepatitis B cases by occupation and age group, 2005–2013.

Most of those in the Worker class who contracted the HBV virus were aged 20–39 (Fig. 4).

### Regional distribution

**Figure 5:**
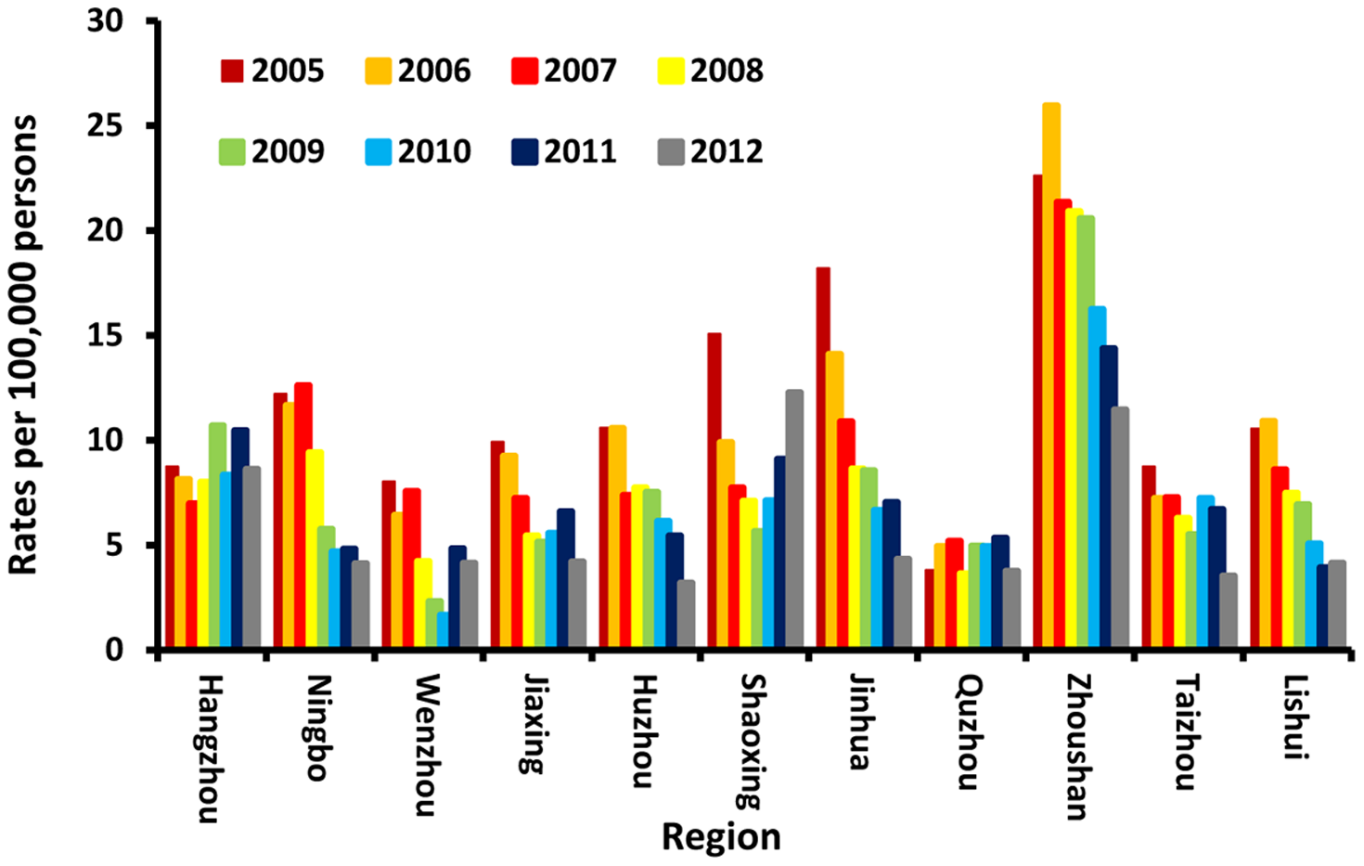
Reported rates of acute hepatitis B cases by region, 2005–2012.

Notification rates of acute hepatitis B cases from 2005 through 2012 are shown by region in Fig. 5. Data for 2013 were not available.

Zhoushan, Zhejiang's only the island city, showed rates twice as high as the mainland (Fig. 5). In 2005, 2006, 2007, 2008, 2009, 2010, 2011, and 2012 Zhoushan city reported 22.60, 25.95, 21.33, 20.89, 20.57, 16.22, 14.36, and 11.44 cases per 100,000 persons, respectively. So, despite their higher rate of infection, notification rates in Zhoushan City did decline at about the same pace (from 22.60 cases in 2005 to 11.44 cases in 2012) as mainland rates (from 10.87 to 5.56).

## Discussion

The authors found no previous research classifying notification rates of acute hepatitis B by age, gender, occupation, or region.

This study determined that, after the implementation of a series of prevention and control strategies, the secular trend on notification rates of acute hepatitis B cases from 2005 through 2013 were significantly dropped among the general population. Significantly economic development and improved sanitary conditions in Zhejiang Province, of course, are also likely to have contributed to these declining rates [8].

The most dramatic decline occurred among people aged ≤19 years. This decline correlates with and highlights the benefits of this massive child-vaccination strategy which China's public health agency so successfully introduced [9]–[10]. This measurable reduction of HBV infection among children is consistent with immunization results in industrialized areas as well as in a few less-developed countries [11]–[14]. For example, during Taiwan's 1984–1999 universal infant immunization, the HBsAg positivity rate among children decreased from 9.8% to 0.7% [12]. In Gambia, from 1986 to 1999, during massive routine vaccination, HBsAg-positive seroprevalence in children decreased from 10% to 0.6% [13]. This 2005–2013 study of Zhejiang Province provides another example of preventing and controlling hepatitis B in children through universal infant vaccination.

The interpretation of acute hepatitis B notification rates is notably hampered by a lack of information. Since most newly infected people are asymptomatic, they are not aware of their infection, so they fail to seek medical care. The infected sub-groups are less likely to be reported [15], so their acute hepatitis B incidence may be underreported.

Hepatitis B is still a major public problem for the Zhejiang region. Although reported rates of acute hepatitis B cases decreased between 2005 and 2013, a recent seroepidemiological survey conducted among a large number of representative participants in Zhejiang Province showed the current prevalence of HBsAg stands at 6.13% [8], thus defining Zhejiang as having intermediate HBV endemicity [5], which is consistent with the previous report [3]. The national seroprevalence survey conducted in 2006 showed that China was an intermediated HBV endemicity [3]. The same report also showed that, compared with the national serosurvey in 1992, the prevalence of HBsAg among children aged 1–14 years born after the start of the hepatitis B vaccination programme was lower than the same age group. However, the prevalence of adults aged 20–59 years was similarly high in both surveys. These trends were also observed in eastern (Zhejiang Province included) and western China. It reflected that routine childhood immunization had an important effect on controlling and preventing HBV for children. Though comparison of the rate between Zhejiang Province and western provinces was hampered by the study design and the surveillance quality, seroprevalence study in 2006 showed that HBsAg positive rate for the general population in eastern China (6.5%) was lower than that of western China (8.3%) [3]. Further study is needed to investigate this issue.

Analysis by region shows that the immunization program was implemented effectively in Zhejiang Province. The average reportable hepatitis B incidence dropped about 50% between 2005 and 2012. However, notification rates differed by region and year. The highest region is Zhoushan City, the only island of Zhejiang Province. This result is consistent with the previous seroepidemiological study conducted in the same region in 2013 [16]. Further study needs to focus on why people in the island are more susceptible to hepatitis B than those on the mainland.

The notification rate among the Worker class went up from 72.02% in 2005 to 77.45% in 2013. This 5.43% gain showed that the program was least successful among this lower socio-economic Worker class. In addition, the rate was statistically significantly higher for Worker males than for males in other occupations. One reason for higher rates among these “Worker males” may be that in lower income households, men usually work away from home for long periods while women often stay home to care for the children. The NNDRS does not report risk factors related to acute HBV infection for these “Worker males.” This lack of solid information implies a need for the NNDSS to develop ways of identifying risk factors related to acute hepatitis B infection. We can consider some factors related with high-risk behaviors for them to be infected with HBV. For instance, a) sharing contaminated blades and toothbrushes in barber shops and crowded accommodations, b) invasive medical or dental treatment with contaminated instruments, or c) multiple sexual partners [5], [16]. Further study is needed, however, to analyze the effect on immunization strategy after controlling the confounding factor such as social-economic status.

The notification rate of acute hepatitis B cases was greatest for those aged 20–39, which is consistent with a previous study [17]. One reason their rate is high may be that they did not receive hepatitis B vaccine under the nationwide vaccination policy. So they have almost no protective antibodies against hepatitis B. Another cause of their high rate, however, may be that their social activity and sexual frequency peaked during young adulthood [18].

In 1992, the national seroepidemiological study showed that the highest rate of HBsAg positivity was in children aged <15 years. Yet the current study shows that, from 2005 through 2013, the highest rate was in young adults. These findings reveal a definite shift in the hepatitis B transmission route. In the 1990s the primary route was vertical, mother-to-child. The current route, however, is horizontal, adult-to-adult.

The vaccination of young adults has been largely neglected because the universal routine vaccination schedule focuses on children. Since 2010, hepatitis B vaccine has been recommended for those adults who are at high risk of HBV infection. But, vaccination for children is mandatory, vaccination for adults is merely voluntary, so not enough adults receive HBV vaccine. If adults hope to be immunized, they have to go to a vaccination clinic. Yet few adults are motivated to seek out a clinic, because they seldom receive information about the dangers or preventions of hepatitis B.

Children, on the other hand, have much more chances to be immunized with hepatitis B vaccine than adults. If a child has not been immunized, an EPI staff member calls the parents or sends a text message. As a result, approximately 96% of Zhejiang Province children were vaccinated against hepatitis B in 2013 while less than 10% of adults were vaccinated [19].

This lack of focus on the group with the highest rate of infection presents the need for a new strategy to control and prevent the horizontal transmission of hepatitis B among young adults. We therefore recommend a universal HBV vaccination programme aimed at pre-adults. This programme should be implemented and strengthened among high school students, tertiary institutions students, and army recruits.

## Conclusions

The secular trend of notifiable rates of acute hepatitis B infection in southern China is decreasing. The primary hepatitis B transmission route has shifted from the vertical mother-to-child mode to the horizontal adult-to-adult mode. The current routine vaccination policy, however, focuses largely on vertical (mother-to-child) transmission. Therefore the vaccination strategy needs to focus also on those young adults who are at risk of contracting the hepatitis B virus.

## Supporting Information

S1 Figure **Reported rates of acute hepatitis B cases by gender and year, 2005–2013.**
(TIF)

S1 Table **Interaction analysis on gender and occupation.**
(DOCX)
