# Supplementary figures and images for: Trends of Acute Hepatitis B Notification Rates in Eastern China from 2005 to 2013
Source: PLoS One. 2014 Dec 12;9(12):e114645. doi: 10.1371/journal.pone.0114645 (PMC4264791; doi:10.1371/journal.pone.0114645)

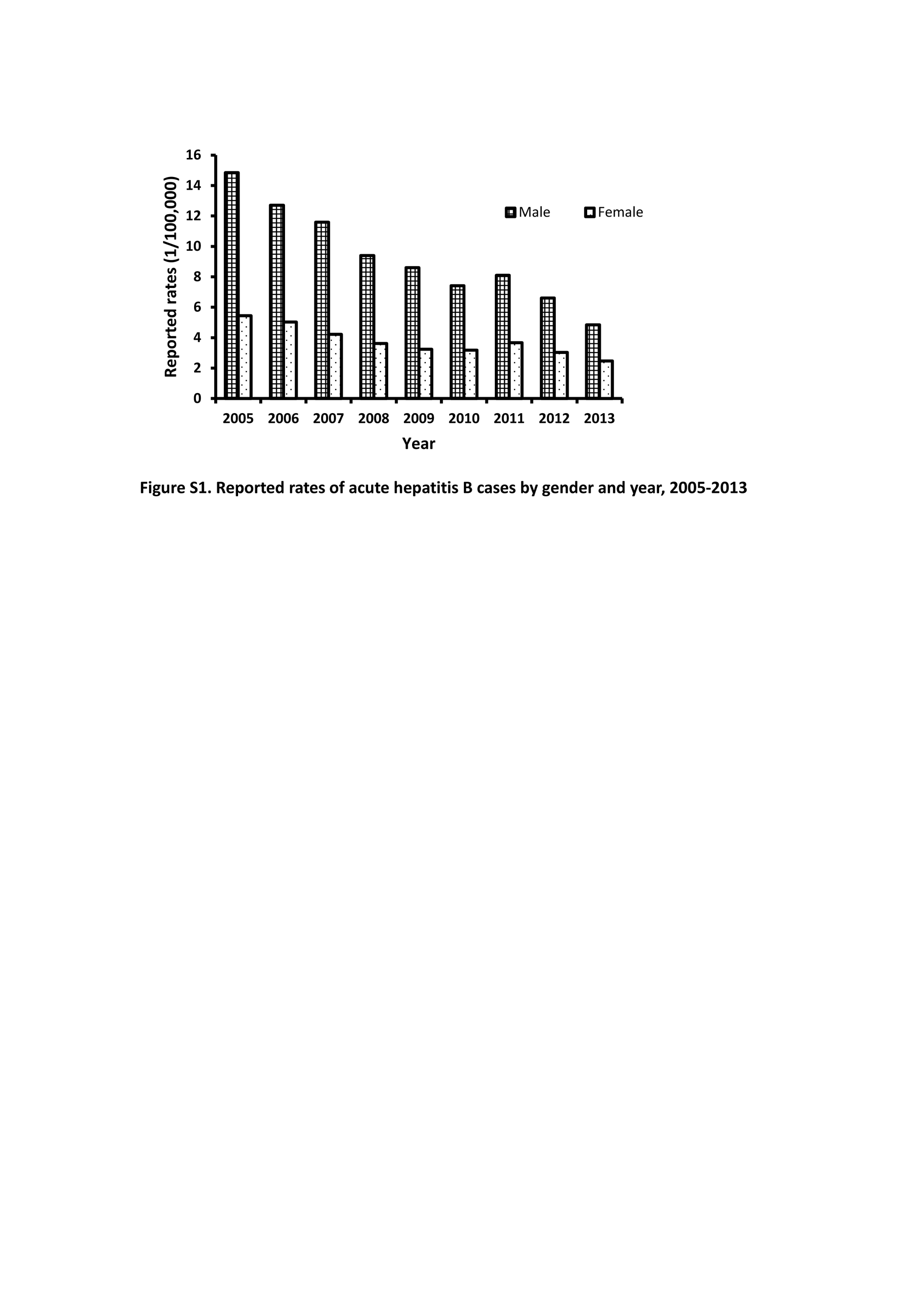

Supplement: S1 Figure — Reported rates of acute hepatitis B cases by gender and year, 2005–2013. (TIF) [file pone.0114645.s001.tif]
