# Supplementary material for: Trends of Acute Hepatitis B Notification Rates in Eastern China from 2005 to 2013
Source: PLoS One. 2014 Dec 12;9(12):e114645. doi: 10.1371/journal.pone.0114645 (PMC4264791; doi:10.1371/journal.pone.0114645)
Supplement: S1 Table — Interaction analysis on gender and occupation. (DOCX) [file pone.0114645.s002.docx]

Table S1. Interaction analysis on gender and occupation

|  | Ratio of different occupation (%) | | | | | |
| --- | --- | --- | --- | --- | --- | --- |
| Gender | Worker | Commercial | Cadres | Personnel | Other | Total |
| Males | 50.8* | 3.6 | 3.1 | 1.0 | 13.5 | 72.0 |
| Females | 20.8 | 1.3 | 0.7 | 0.8 | 4.4 | 28.0 |
| Total | 71.6 | 5.0 | 3.9 | 1.8 | 17.9 | 100.0 |

*P=0.000; χ^2^=165.
